# Exploring attractor bifurcations in Boolean networks

**DOI:** 10.1186/s12859-022-04708-9

**Published:** 2022-05-11

**Authors:** Nikola Beneš, Luboš Brim, Jakub Kadlecaj, Samuel Pastva, David Šafránek

**Affiliations:** Faculty of Informatics, Masaryk University, Brno, Czechia

**Keywords:** Boolean networks, Attractor bifurcation, Symbolic computation, Software tool, type-1 interferons

## Abstract

**Background:**

Boolean networks (BNs) provide an effective modelling formalism for various complex biochemical phenomena. Their long term behaviour is represented by attractors–subsets of the state space towards which the BN eventually converges. These are then typically linked to different biological phenotypes. Depending on various logical parameters, the structure and quality of attractors can undergo a significant change, known as a bifurcation. We present a methodology for analysing bifurcations in asynchronous parametrised Boolean networks.

**Results:**

In this paper, we propose a computational framework employing advanced symbolic graph algorithms that enable the analysis of large networks with hundreds of Boolean variables. To visualise the results of this analysis, we developed a novel interactive presentation technique based on decision trees, allowing us to quickly uncover parameters crucial to the changes in the attractor landscape. As a whole, the methodology is implemented in our tool AEON. We evaluate the method’s applicability on a complex human cell signalling network describing the activity of type-1 interferons and related molecules interacting with SARS-COV-2 virion. In particular, the analysis focuses on explaining the potential suppressive role of the recently proposed drug molecule GRL0617 on replication of the virus.

**Conclusions:**

The proposed method creates a working analogy to the concept of bifurcation analysis widely used in kinetic modelling to reveal the impact of parameters on the system’s stability. The important feature of our tool is its unique capability to work fast with large-scale networks with a relatively large extent of unknown information. The results obtained in the case study are in agreement with the recent biological findings.

## Background

*Boolean networks* [1] represent a well-established modelling formalism commonly used to study complex biological systems, such as gene regulatory networks. The simple underlying structure of a Boolean network (BN) combined with its expressiveness makes it particularly well suited for in silico analysis using formal methods. Consequently, we see a rapid development of new methods in this area [2–4]. A more thorough overview of BN methods, models and tools for computational systems biology is then available in, e.g., [5].

A critical element of BN modelling is the structure of the network *attractors*. Attractors correspond to the long-term behaviour of the BN. Informally, an attractor is a connected subset of the network states in which the system, if left unperturbed, stays forever. Attractors then manifest in biologically relevant *phenotypes*, such as differentiated cell types and tissues [6], or biological rhythms and sustained oscillations [7]. However, the structure of network attractors is closely related to the employed variable updating scheme [8].

A BN consists of a collection of Boolean variables, the state of which is determined by other variables in the network using associated Boolean *update functions* (one for every variable). We speak about *synchronous dynamics* if all update functions are applied simultaneously at each time point. If only one of the update functions is chosen non-deterministically to modify the corresponding Boolean variable, we speak of *asynchronous dynamics*.

In general, the analysis of synchronous dynamics is considered more accessible; in the asynchronous case, attractor identification is complicated by the non-deterministic nature of the state transitions. Unfortunately, it is known that the synchronous update can produce unrealistic attractors [9, 10]. However, models with asynchronous update can cover the real attractors quite well, though it has been recently shown that some exceptions exist [2]. Our method, therefore, primarily focuses on asynchronous dynamics.

In asynchronous systems, we generally recognise three distinct types of attractors corresponding to different long-term behaviour. Briefly, the first case is when the network evolves to a single stable state. Such states are the fixed points or *point attractors*. The second situation is when the network periodically oscillates through a finite sequence of states-an *oscillating attractor* (the discrete equivalent of a limit cycle in continuous systems). The third situation is classified as a so-called *disordered attractor* (sometimes also called a *complex* attractor). Such an attractor is neither stable nor periodically oscillating. The system may behave unpredictably due to the non-determinism of the asynchronous dynamics. Aside from these structural characteristics, a particular attractor’s phenotype may also be associated with specific values of relevant network variables.

One particular problem when detecting attractors of the network (and their associated phenotypes) is that the update functions of the BN are often only partially known or may be subject to influence from external factors. In such a case, we speak of *parametrised Boolean networks* [11], in which the update functions depend on a set of *logical parameters*.

In parametrised BNs, the attractors change as the values of the parameters are varied. Some of these changes might lead to qualitatively different attractors (i.e. variation in the count and/or types of attractors). Such a qualitative change is called a *bifurcation*. To study bifurcations in discrete systems, we can classify the parameter valuations into distinct *classes* based on qualitatively different types of behaviour. We call such classification a *bifurcation function* and refer to the process of computing and studying this function as *attractor bifurcation analysis* [12]. As such, bifurcation analysis is one of the most fundamental approaches that allow exploring the behaviour of biological networks on a global scale.

The core problem of bifurcation analysis, i.e. attractor detection, has been widely studied before in different contexts, but primarily in *non-parametrised* BNs (see [13] for an overview). Known attractor detection methods can be used for bifurcation analysis by employing a naïve parameter scan. However, such approach does not scale to systems with a large number of logical parameters, as the size of the parameter space rises exponentially with the number of parameters.

For synchronous systems, there are efficient exact solutions for attractor detection in terms of SAT [14, 15], constraint programming [16], integer-programming [17], or BDD-based representation [8, 18]. In the asynchronous cases, various techniques have been employed, including BDDs [8, 19], optimisation [20], algebraic methods [21], SAT [22], answer set programming [23], concurrency theory [24], sampling [25], or network structure decomposition [26].

Alternatively, algebraic methods based on semi-tensor products  [27] and polynomial matrix representations  [28] can be employed for various analysis tasks in synchronous BNs  [29–31]. However, these methods ultimately suffer from the curse of dimensionality, since the dimension of the state-transition matrix grows exponentially with the number of variables in the BN  [32].

Several computational tools have been developed to construct, visualise, and analyse attractors in non-parametrised BNs. Amongst them, the established tools include ATLANTIS [33], Bio Model Analyzer (BMA) [34], BoolNet [35] and ViSiBooL [36], PyBoolNet [37], lnet [38], The Cell Collective [39], CellNetAnalyzer [40], and ASSA-PBN [41]. Furthermore, tools targeting parameter synthesis in BNs are relevant here as well. These cannot construct the full classification automatically but can often at least identify subsets of parametrisations based on a particular type of long-term behaviour. GINsim [42] supports parameter synthesis of both synchronous and asynchronous models indirectly through an external model checker (however, the model has to be manually parametrised). Finally, TREMPPI [43] is an online tool providing parameter synthesis of asynchronous models w.r.t. LTL.

To the best of our knowledge, the only existing methodology targeting the concept of bifurcation analysis in Boolean networks has been proposed in  [44, 45]. In particular, Abou-Jaoudé et al. adapt the notion of *bifurcation diagrams* to the settings of logical models, which also covers Boolean networks. This notion is established by relating the discrete dynamics of the logical model with the continuous dynamics of a corresponding piecewise differential model. The actual bifurcation diagram then displays attractors of the network along a growing sequence in the lattice of the model parameters.

However, it is worth noting that the number of such sequences tends to be very large in non-trivial BNs. Additionally, each such sequence only covers a relatively small portion of the parameter space. Consequently, many different bifurcation diagrams may be necessary to fully characterise the long-term behaviour of a single network. Meanwhile, our novel approach encodes this information within a single structure: a bifurcation decision tree. Additionally, our approach is supported by efficient symbolic algorithms. As we demonstrate in our results, this allows us to efficiently handle realistic large-scale networks, which, to the best of our knowledge, is not currently possible within the framework of  [44].

In this paper, we present a comprehensive methodology for automated attractor bifurcation analysis of parametrised BNs, fully implemented in our tool AEON [46, 47]. The work is based on our previous research in this area [12, 48–51], in particular [12]. The main contribution is in combining the theoretical concepts into a singular methodology. In addition, this work addresses two remaining key challenges in attractor bifurcation analysis of BNs:

1. A concise specification of partially unknown parametrised BNs using *uninterpreted Boolean functions*, including static validation of such parametrised models.
2. Extending our previous work on the visualisation of bifurcation functions using decision trees [12], we introduce a novel language of node attributes representing hierarchically the parameter constraints affecting the parametrised update functions.

Additionally, we introduce an interactive decision tree editor in AEON, which utilises the above mentioned improvements to facilitate a user-friendly experience. In part of these improvements, we have also incorporated a comprehensive stability analysis workflow into the decision tree editor. Finally, we demonstrate the presented methodology on a complex human cell signalling network describing the activity of type-1 interferons and related molecules interacting with the SARS-COV-2 virion, which, to the best of our knowledge, has not been studied in this way before.

## Preliminaries

We start by formally introducing the modelling framework of parametrised Boolean networks and the notion of attractor bifurcation in such models. More technical details about this topic are discussed in [12] and [46].

### Boolean networks

**Fig. 1:**
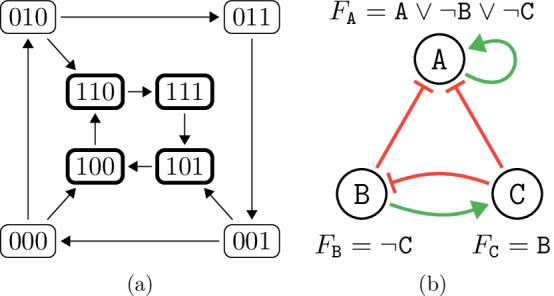
(**a**) The asynchronous state-transition graph of the Boolean network in Example 1. Each state specifies the values of the network variables in the lexicographic order. The highlighted states show an oscillating attractor of the network. (**b**) The regulatory graph and update functions of the Boolean network in Example 1. Green and red arrows represent activating and inhibiting regulations, respectively. A solid arrow implies that the regulation is essential in the corresponding update function

We consider the standard (non-parametrised) *Boolean network* (BN) to be given as a regulatory graph of Boolean variables, where each variable has an associated Boolean update function. Formally, we have a finite set of Boolean variables ${\mathcal {V}}$ (denoted $\mathtt {A}, \mathtt {B}, \ldots$), regulations $R \subseteq {\mathcal {V}}\times {\mathcal {V}}$, and a family of Boolean *update functions* ${\mathcal {F}} = \{ F_{\mathtt {A}} \mid \mathtt {A} \in {\mathcal {V}}\}$. The signature of each $F_{\mathtt {A}}$ is determined by the *regulatory context* ${\mathcal {C}}(\mathtt {A}) = \{ \mathtt {B} \mid (\mathtt {B}, \mathtt {A}) \in R \}$. Specifically, $F_{\mathtt {A}} : \{ 0, 1 \}^{{\mathcal {C}}(\mathtt {A})} \rightarrow \{ 0, 1 \}$. Here, the members of ${\mathcal {C}}(\mathtt {A})$ are called *regulators* of $\mathtt {A}$, and $\mathtt {A}$ is then referred to as the regulation *target*.

The network’s behaviour is represented by its *asynchronous state-transition graph*. *State* *s* of a BN assigns each variable a Boolean value, i.e. $s: {\mathcal {V}}\rightarrow \{0, 1\}$. Graph’s vertices are then the $2^{|{\mathcal {V}}|}$ possible states of the BN. The edges of the graph correspond to the asynchronous application of the update functions to the current state. Formally, for every $s \rightarrow t$ in the graph, there exists a variable $\mathtt {A} \in {\mathcal {V}}$ such that $t(\mathtt {A}) = F_{\mathtt {A}}(s)$ and the remaining $\mathtt {B} \in {\mathcal {V}}$ are unchanged (i.e. $s(\mathtt {B}) = t(\mathtt {B})$).

#### Example 1

Consider a simple BN with ${\mathcal {V}}= \{ \mathtt {A}, \mathtt {B}, \mathtt {C} \}$ such that ${\mathcal {C}}(\mathtt {A}) = \{ \mathtt {A}, \mathtt {B}, \mathtt {C} \}$, ${\mathcal {C}}(\mathtt {B}) = \{ \mathtt {C} \}$, and ${\mathcal {C}}(\mathtt {C}) = \{ \mathtt {A} \}$. Additionally, let $F_{\mathtt {A}} = \mathtt {A} \vee \lnot \mathtt {B} \vee \lnot \mathtt {C}$, $F_{\mathtt {B}} = \lnot \mathtt {C}$, and $F_{\mathtt {C}} = \mathtt {B}$. The state-transition graph of this network is shown in Fig. 1 (a).

Notice that the dependence between variables is typically monotonous. For example, an increase in $\mathtt {B}$ cannot decrease $\mathtt {C}$; hence we might say that $\mathtt {B}$ regulates $\mathtt {C}$ positively. We often graphically represent a BN using its regulatory graph in which we include these observations. For the network in Example 1, such a graph is shown in Fig 1b, with positive regulations (*activations*) using green and sharp arrow tips, while negative regulations (*inhibitions*) use flat red arrows.

Furthermore, for every $(\mathtt {B}, \mathtt {A}) \in R$, in terms of [52], every regulator $\mathtt {B}$ is *essential* (also *observable*) in $F_{\mathtt {A}}$. The essentiality of regulators mandates that whenever $\mathtt {B}$ regulates $\mathtt {A}$, $\mathtt {B}$ needs to have a measurable impact on the value of $F_{\mathtt {A}}$. Formally, there is a state *s* such that flipping the value of $\mathtt {B}$ in *s* also flips the value of $F_{\mathtt {A}}(s)$.

However, we can further generalise this notion of essentiality. Consider the update function $F_{\mathtt {A}} = \mathtt {A} \vee \lnot \mathtt {B} \vee \lnot \mathtt {C}$. When $\mathtt {A} = 1$, the value of $F_{\mathtt {A}}$ does not depend on either $\mathtt {B}$ or $\mathtt {C}$. Hence $\mathtt {B}$ and $\mathtt {C}$ are not essential in $F_{\mathtt {A}}$ for $\mathtt {A} = 1$ (however, they are both essential for $\mathtt {A} = 0$). In this fashion, we can list an arbitrary partial state of the BN to further specify the dependence between two variables. For example, we can say that $\mathtt {C}$ is essential in $F_{\mathtt {A}}$ for $\mathtt {A} = 0$ and $\mathtt {B} = 1$. That is because there is a state *s* which satisfies this partial assignment (i.e. $s(\mathtt {A}) = 0$ and $s(\mathtt {B}) = 1$), and flipping the value of $\mathtt {C}$ in *s* flips the value of $F_{\mathtt {A}}(s)$. This notion of generalised essentiality will come into play later for parametrised Boolean networks when we discuss how AEON visualises the space of possible update functions.

### Network attractors

In practice, a crucial question of BN modelling is what eventually happens to the network state in the long term. This information can be obtained by studying the network’s *attractors*. These correspond to the bottom (also terminal) strongly connected components (BSCC) of the state-transition graph. Assuming no transition is delayed indefinitely, these are the regions of the state space where the network eventually stays forever.

Formally, a BSCC is a maximal set of states *S* such that for all $s \in S$, the states reachable from *s* are exactly the set *S*. Consider the network from Example 1 and its state-transition graph in Fig. 1a. It has one attractor, namely, the set $\{ 100, 110, 111, 101 \}$. Once this set is reached, the network oscillates between these four states forever. In practice, attractors can exhibit different types of behaviour:

- *Stability* ($\odot$) An attractor is stable if it consists of a single state. The network then stays in this state forever. Sometimes, this type of attractor is also called *equilibrium* or *sink*.
- *Oscillation* ($\circlearrowleft$) A single cycle of states, such as in our example, is called an oscillating attractor. The length of such a cycle is its *period*. These attractors are also sometimes called *limit cycles* or *periodic* attractors.
- *Disorder* ($\rightleftarrows$) Finally, an attractor is disordered (also *aperiodic*) if it is neither stable nor oscillating. Even though the network will stay in the attractor forever, it will behave somewhat unpredictably due to the non-determinism of the asynchronous state-transition graph.

Since the network will often have multiple attractors, its long-term behaviour is characterised by a multi-set over these three attractor types $\{ \odot , \circlearrowleft , \rightleftarrows \}$. We call such multi-set a *behaviour class*, and we denote the set of all possible behaviour classes ${\mathfrak {C}}$. In Example 1, the network’s behaviour class is only one $\circlearrowleft$ attractor.

Observe that this notion of behaviour classes follows intuitively from the established bifurcation analysis methodology in continuous dynamical systems  [53]. In the continuous case, attractors are differentiated based on their topological properties in the continuous state space. This distinction naturally leads to the recognition of stable equilibria (single-state attractors), limit cycles (oscillating attractors), and chaos (attractors consisting of disordered trajectories). However, note that the workflow is (to some extent) modular in this regard. If a different classification is desired, assuming a symbolic algorithm to perform this classification based on attractor states exists, it can be used to supplement or completely replace our proposed classification.

### Parametrised Boolean networks

**Fig. 2:**
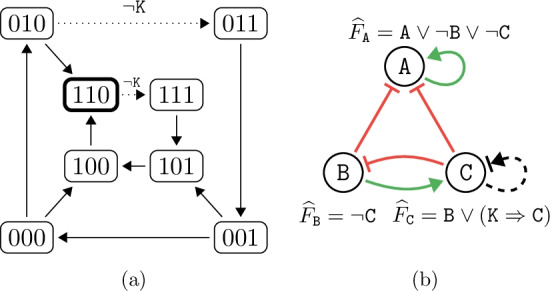
(**a**) The asynchronous state-transition graph of the network in Example 2. The two dotted edges are only present when $\mathtt {K} = 0$. Consequently, the attractor of the network changes to the single highlighted state when $\mathtt {K} = 1$. (**b**) Regulatory graph and parametrised update functions of the Boolean network in Example 2. Compared to Example 1, there is one new regulation, which may not be essential (thus uses a dashed arrow)

Inferring the exact update functions from experimental data is a complex and error-prone task, which often cannot be performed exactly. This uncertainty can be expressed using *logical parameters*, leading us to the introduction of *parametrised Boolean networks*.

A parametrised Boolean network has an associated finite set of *parameter names* ${\mathcal {P}}$ (disjoint with ${\mathcal {V}}$), which parametrise each update function. We use the ${\widehat{F}}_{\mathtt {A}}$ notation for such update functions to differentiate them from their non-parametrised counterparts. The type of each such function is then ${\widehat{F}}_{\mathtt {A}} : \{0, 1\}^{{\mathcal {C}}(\mathtt {A}) \cup {\mathcal {P}}} \rightarrow \{ 0, 1 \}$. Hence to obtain the final result, values of both regulators and parameters are considered.

We call each assignment $p: {\mathcal {P}}\rightarrow \{0,1\}$ a *parametrisation*. With each parametrised Boolean network, we also associate a set of valid parametrisations *P*. This set allows us to arbitrarily restrict which parametrisations are considered admissible for the given network. By fixing one such parametrisation $p \in P$, we obtain a standard non-parametrised Boolean network called a *p*-*instantiation*.

In the worst case, the number of unique instantiations can be doubly exponential in the size of ${\mathcal {V}}$ [54]. It is, therefore, necessary to restrict the set *P* as much as possible. To do this, we often utilise the monotonicity and essentiality properties that we discussed in relation to the non-parametrised BNs.

Specifically, when we say a regulation is monotonous in a parametrised BN, we mean that for every $p \in P$, the *p*-instantiation has this property. Consequently, we can use the visual elements we introduced for non-parametrised regulatory graphs in a parametrised setting as well.

In a non-parametrised BN, we assumed each regulation was essential. In a parametrised BN, we pose no such requirement, as an unknown update function need not always depend on all regulators. Instead, we assume each regulation marked as essential (solid arrow) must be essential in each instantiation, and regulations not denoted as such (dashed arrows) may or may not be employed by valid instantiations.

#### Example 2

Let us now consider Example 1, but extended to a parametrised Boolean network in the following way: We add a new regulation $(\mathtt {C}, \mathtt {C})$ without any assumptions about monotonicity or essentiality (drawn in black, with a combined sharp and flat arrow tip). Then, let ${\mathcal {P}}= \{ \mathtt {K} \}$ and ${\widehat{F}}_{\mathtt {C}} = \mathtt {B} \wedge (\mathtt {K} \Rightarrow \mathtt {C})$. As valid parametrisations *P*, we naturally consider both $\mathtt {K} = 0$ and $\mathtt {K} = 1$. Consequently, $\mathtt {K}$ effectively switches ${\widehat{F}}_{\mathtt {C}}$ between $\mathtt {B}$ (as used in Example 1) and $\mathtt {B} \wedge \mathtt {C}$. Fig. 2 shows how this influences the state-transition graph and the regulation graph.

As we see in Fig. 2a, even a small change in an update function can drastically alter the attractors of a Boolean network. Knowing when such change occurs and which parametrisations produce similar behaviour is the motivation behind the attractor bifurcation problem.

### Attractor bifurcation in Boolean networks

Given a parametrised Boolean network with a set of valid parametrisations *P*, the goal of attractor bifurcation analysis is to compute the *bifurcation function* ${\mathcal {A}}: P \rightarrow {\mathfrak {C}}$. For a valid parametrisation $p \in P$, the function ${\mathcal {A}}$ provides the behaviour class (i.e. multiplicity of different attractor types) of the corresponding *p*-instantiation.

Note that such ${\mathcal {A}}$ is not concerned with the exact states of individual attractors but instead provides a very general overview (in terms of stability, oscillation and disorder) of what is admissible within the given network. In some instances, further investigation into the internal structure of the attractors within each behaviour class may be beneficial (as is also the case for bifurcation analysis in continuous systems). We address this aspect in more detail later in the text.

Overall, we believe bifurcation analysis to be an excellent tool for high-level analysis of the parameter space, which can then guide further experiments. Additionally, as we show in this case study, it can help identify critical network parameters that negatively affect the network’s behaviour.

## Methods

Although the bifurcation function contains all important information about the behaviour classes of a given network, creating a large parametrised network and then inspecting the impact of parameters on its behaviour is still a challenging task. In this section, we discuss how our tool, AEON, addresses these challenges and what new features this adds to Boolean network modelling.

Our contribution is the following: First, to make the specification of a parametrised network more natural, AEON models support uninterpreted Boolean functions inside update functions. Using these large, partially unknown update functions can be constructed much more concisely. Then, thanks to its use of advanced symbolic algorithms, AEON can compute the bifurcation function for systems with tens or even hundreds of variables and parameters. Finally, using its unique interactive decision tree visualisation, AEON enables the discovery of parameters detrimental to the system’s behaviour.

### Uninterpreted Boolean functions

In AEON, a network can declare an arbitrary number of *uninterpreted Boolean functions* $\mathtt {K}^{(a)}$, $\mathtt {L}^{(b)}$, $\mathtt {M}^{(c)}$, etc. Here, *a*, *b*, *c* are the function arities, which can be omitted when clear from the context. Such functions are similar to the standard parameters discussed in the previous section in that they represent an unknown but fixed part of the network update function. In fact, an uninterpreted function of arity 0 is equivalent to a parameter.

However, uninterpreted functions can be often used to express uncertainty much more naturally. Consider the update function ${\widehat{F}}_{\mathtt {A}} = \mathtt {A} \vee \lnot \mathtt {B} \vee \lnot \mathtt {C}$ from our running example. In a situation where the dependence of $\mathtt {A}$ on $\mathtt {B}$ is certain, but the exact relationship between $\mathtt {A}$ and $\mathtt {C}$ in the equation is unknown, we can introduce an uninterpreted function $\mathtt {K}^{(2)}$ and write ${\widehat{F}}_{\mathtt {A}} = \lnot \mathtt {B} \vee \mathtt {K}(\mathtt {A}, \mathtt {C})$. In turn, AEON will consider all admissible binary functions in place of $\mathtt {K}$ as network parametrisations.

Internally, every such function $\mathtt {K}$ of arity *i* is represented using $2^{i}$ standard Boolean parameters; one for each row of the function table of $\mathtt {K}$. Since model analysis is performed symbolically using binary decision diagrams [55], AEON can map between applications of update functions and symbolic constraints on the network parameters. As a result, the asynchronous state-transition graph can be explored without explicitly enumerating either the network states or parametrisations.

Finally, to limit the space of valid parametrisations, AEON allows the user to specify which regulations are monotonous or essential. Any instantiation that does not meet these criteria is then excluded from the analysis. For example, in the case of ${\widehat{F}}_{\mathtt {A}} = \lnot \mathtt {B} \vee \mathtt {K}(\mathtt {A}, \mathtt {C})$, we know that both $\mathtt {A}$ and $\mathtt {C}$ are essential, with $\mathtt {A}$ being activating and $\mathtt {C}$ inhibiting. This leaves only two valid instantiations of $\mathtt {K}$, namely $\mathtt {A} \wedge \lnot \mathtt {C}$ and $\mathtt {A} \vee \lnot \mathtt {C}$.

This type of analysis is performed even for fully specified update functions. As a result, AEON can be used to validate that the regulatory graph and update functions follow the same properties. While this benefit may seem trivial, when developing AEON, we discovered many publicly available models with inconsistencies between their regulatory graphs and their update functions.

Such parametrised Boolean networks with uninterpreted functions can be developed in an interactive online editor, which is a part of AEON. This network editor also performs integrity checks between the update functions and the regulatory graph on the fly, ensuring the model is always consistent.

### Bifurcation decision trees

**Fig. 3:**
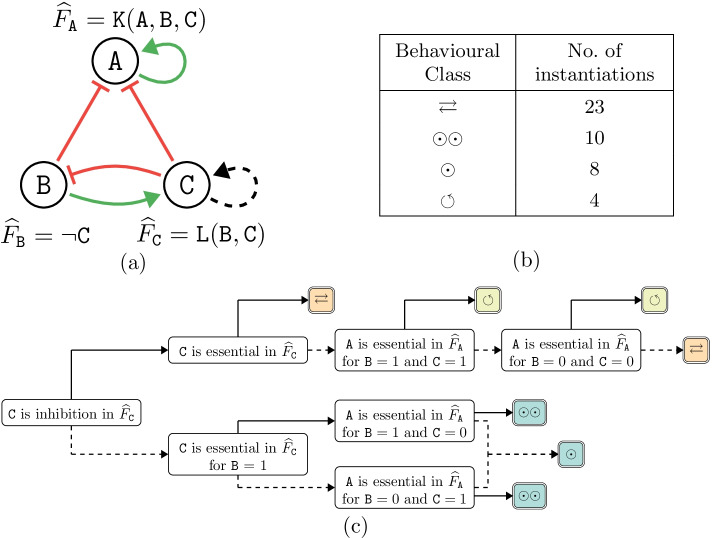
(**a**) Regulation graph of the network in Example 3. (**b**) Overview of the bifurcation function in Example 3 as computed by AEON. (**c**) A bifurcation decision tree constructed in AEON with four possible behavioural classes ($[\odot ], [\circlearrowleft ], [\rightleftarrows ]$, and $[\odot , \odot ]$) for the network in Example 3. Solid and dashed arrows represent positive and negative decisions, respectively

When given a parametrised model, AEON symbolically computes the attractors for all valid parametrisations. Based on these attractors, AEON assigns each parametrisation its behaviour class from ${\mathfrak {C}}$. Initially, this bifurcation function can be displayed as a simple table with the option to obtain witness instantiations for each discovered behaviour class and inspect their attractor state space.

#### Example 3

Let us consider a different parametrised variant of the Boolean network from Example 1. We declare two uninterpreted functions, $\mathtt {K}^{(3)}$ and $\mathtt {L}^{(2)}$. We then write ${\widehat{F}}_{\mathtt {A}} = \mathtt {K}(\mathtt {A}, \mathtt {B}, \mathtt {C})$ and ${\widehat{F}}_{\mathtt {C}} = \mathtt {L}(\mathtt {B}, \mathtt {C})$. Set *P* is restricted to the 45 parametrisations that follow the properties of the regulatory graph in Fig. 3 (a). AEON discovered four distinct behaviour classes in the bifurcation function ${\mathcal {A}}$ of this network. An overview of ${\mathcal {A}}$ is shown in Fig. 3b.

However, as we see in Fig. 3b, such tabular representation does not meaningfully convey the relationship between behavioural classes and parametrisations of the network. AEON thus enables the user to explore the bifurcation function more comprehensively using *decision trees*.

Decision trees commonly appear in machine learning [56], where they are used to represent classifiers and decision strategies. A decision tree is a flowchart-like structure in which each node represents a test on some attribute(s), and each leaf represents one class or end result. Furthermore, each leaf can have an assigned confidence level, representing the precision of the result. When used to visualise the bifurcation function, we refer to this data structure as the *bifurcation decision tree* (BDT).

The bifurcation function as stored internally by AEON already has a compact symbolic representation using binary decision diagrams. However, this representation is very hard to read, especially in models with complex uninterpreted Boolean functions, as the decision diagram is essentially a compressed representation of their logical function tables. The purpose of bifurcation decision trees is then to provide an alternative, human-friendly visualisation format.

To this end, we provide a wide range of decision attributes, which the user can interactively select based on their visualisation goal and any prior knowledge of the network. To simplify the choice, the attributes are pre-sorted based on *information gain* [57], a heuristic used in standard machine learning algorithms [56] to greedily select optimal decision attributes.

The attributes considered by AEON fall into the following categories:

- For a basic Boolean parameter (i.e. uninterpreted function of arity 0), simply decide based on the value of such parameter.
- If there is a regulation without monotonicity or essentiality constraint, decide on the possible presence of such constraint. For example, consider how making a regulation positively monotonous influences the behaviour of the network.
- In a parametrised update function, consider possible generalised essentiality constraints as discussed in Section 2.1. For example, consider if the behaviour changes when we require that $\mathtt {C}$ is essential in ${\widehat{F}}_{\mathtt {A}}$ for $\mathtt {A} = 1$.
- Finally, one can decide based on the individual rows of the uninterpreted functions, e.g. test whether $\mathtt {L}(1, 0)$ is 1 or 0. Such attributes are not very human-friendly but can occasionally uncover interesting relationships and are thus included for completeness.

If we load the bifurcation function produced by Example 3 into AEON ’s decision tree explorer, we can produce a visualisation similar to the one in Fig. 3c. From this decision tree, we can quickly observe several interesting properties about the network from Example 3.

First, non-trivial attractors are only present if $(\mathtt {C}, \mathtt {C})$ is a (possibly non-essential) inhibition. Whenever $\mathtt {C}$ has some other than negative effect in ${\widehat{F}}_{\mathtt {C}}$, the network is stable. If $(\mathtt {C}, \mathtt {C})$ is not essential, the network decides between oscillation and disorder based on what happens in ${\widehat{F}}_{\mathtt {A}}$ when $\mathtt {B} = \mathtt {C}$. In the stable branch, the presence of bistable behaviour depends on values of ${\widehat{F}}_{\mathtt {C}}$ when $\mathtt {B} = 1$ and values of ${\widehat{F}}_{\mathtt {A}}$ when $\mathtt {B} \not = \mathtt {C}$. Interestingly, all these conditions identify autoregulation as the main driver of attractor bifurcation in this network.

### Variable stability analysis

So far, we have only distinguished between the behaviour of different attractors on a qualitative level (i.e. stability, oscillation, or disorder). Nevertheless, there may be important biological phenotypes that do not manifest as a qualitative change in the network’s long-term behaviour. For example, the network may exhibit two stable states (under different parametrisations) which differ in the values of certain critical variables. This difference may then result in a different biological phenotype, even though the qualitative long-term behaviour of the network is the same.

Changes on this level are harder to detect fully automatically since different models express phenotypes differently. However, changes in long-term variable expression across parametrisations can be tracked by AEON as well. This can be done globally (across the whole model) or locally, meaning for a specific behaviour class or a node in the afore-mentioned bifurcation decision tree. As such, one can investigate the possible long-term outcomes (variable is always 1, always 0, or switches between 1 and 0) across different subsets of the parameter space.

For the simple network in our running example, we can discover that when the network is bistable, it always switches between $\mathtt {A} = 0$ and $\mathtt {A} = 1$ in the two sink states. Additionally, when the network oscillates, the value of $\mathtt {A}$ is always constant. That is, the oscillating variables are always $\mathtt {B}$ and $\mathtt {C}$. This type of information could be then used to, for example, reprogram the network to avoid this switching behaviour.

## Results

We evaluate the method on a systematic study of a complex human cell signalling network describing the activity of type-1 interferons and related molecules interacting with SARS-COV-2 virion. The network is important in explaining the dynamics of *innate immune response* (IIR) – an important biological phenotype, corresponding to the activity of the immune system, and *inflammation* (INFL)—a biological phenotype causing an overreaction that might cause severe health problems in affected tissues (e.g., cytokine storm in [58]).

### Core model

We have adopted the BN model of interferon type-1 pathways produced by the COVID-19 Disease Map project [59]. The original BN model has been constructed from the openly accessible SBGN curated pathway^1^ by employing the automated inference algorithm CaSQ [60]. CaSQ is producing ready-to-simulate (non-parametrised) BNs where influences affecting a certain reaction are combined using disjunction. Parameters are introduced explicitly to represent input variables.

The original BN model contains 93 variables, 157 regulations, and 18 parameters. It is available as an SBML-qual model (original.sbml) and AEON model (original.aeon) in Additional file 1. The parameters can be classified into three groups: virus proteins, input signals, and drugs (azithromycin and GRL0617). The input signals include virus replication detection, PAMP signalling and TREML4.

### Model reduction

Even though the original model can be analysed using AEON without major problems, there is an unexpectedly high number of distinct steady-state attractors. By inspecting the attractor state space in AEON, we discovered that they often represent the same phenotypes. To ease presentation, we have reduced the number of attractors so that it more closely matches the phenotypes of the network.

The extraneous attractors appear due to a set of three mutually dependent variables, NFKBIA, NFKB-NFKBIA-complex and NFKB1-cell. By analysing these three variables in isolation (i.e. keeping only variables on which the variables in this set depend), we discovered that in approx. half of the parametrisations, the variables in the set become bistable.

However, the rest of the model only depends on NFKB1-cell and is unaffected by bistability. As a result, the amount of steady-state attractors is doubled since, for every distinct attractor phenotype, there are two attractors corresponding to the two possible stable situations reported on variables in the set.

We have, therefore, replaced these three variables with a parameter NFKBIA-NFKB1-component, removing other variables that only influence this complex. As a result, the reduced model over-approximates the bistability of the original model by abstracting the removed variables in terms of new parameters in the parameter space. The reduced model finally contains 87 variables, 146 regulations and 17 parameters. Its full description is again part of Additional file 1.

**Fig. 4:**
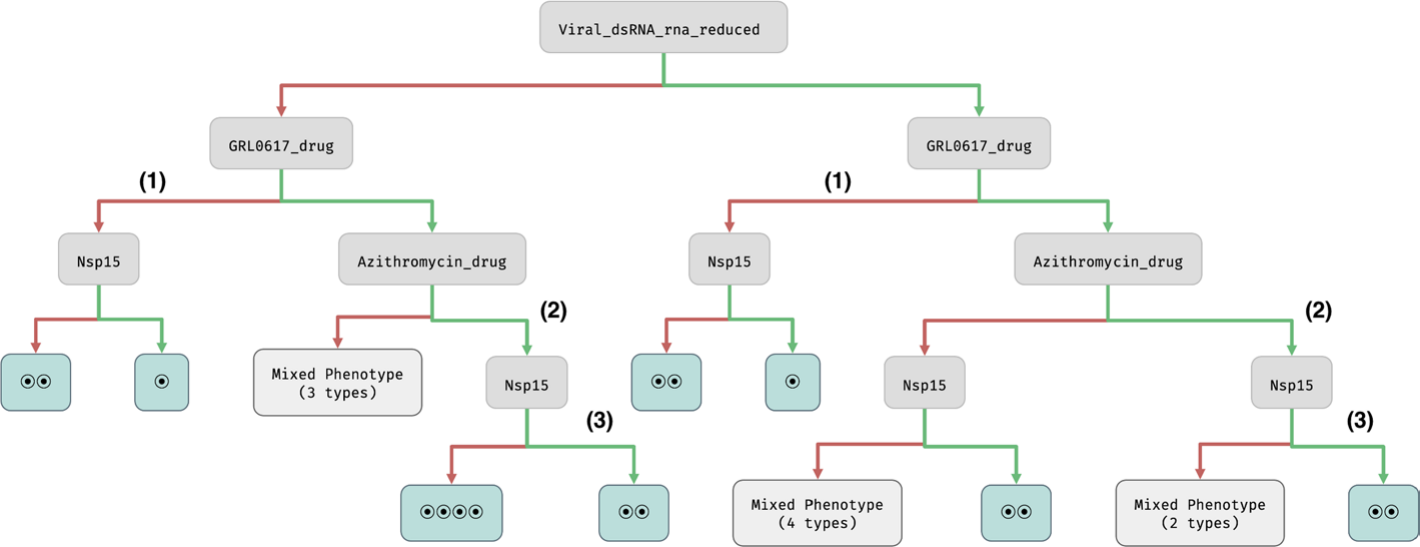
BDT of the reduced model representing the decisions in input variables causing bifurcations and affecting the presence of different attractor phenotypes. The tree is first segmented based on the detection of virus replication by the cell. Then, the decisions marked (1–3) are significant with respect to the studied biological phenotypes: (1) corresponds to the total absence of IIR, (2) shows the interferon production switched off, and (3) makes IIR either off in some instances, or bistable (no instance with IIR positive and stable)

### Model analysis

For in-depth analysis, we use the reduced model. First, we construct the BDT showing the effect of the two suggested drug components on the stabilisation of the interferons systems dynamics. Next, we focus on the distribution of the studied biological phenotypes across the identified attractor phenotypes.

We root the BDT at the input variable representing the presence/absence of the unfolded double-stranded RNA in the cell, indicating the virus is present in the cytosol and is replicating. Fig. 4 shows both possible situations (detected and undetected virus replication). In the next levels of the BDT, we consider the decision points representing the application of the drugs. At first, the GRL0617 is considered due to the fact that it brings the most significant information gain towards the attractor bifurcation (even more significant than most of the virus proteins). Application of azithromycin affects the bifurcation only when used additionally with GRL0617.

In further levels of the BDT, the effect of transcribed virus proteins is unfolded. The most significant information gain is reported for Nsp15. The presence of active Nsp15 in cells under the drug therapy implies bistability. This situation is not affected by virus replication detection. In non-medicated cells, the presence of Nsp15 stabilises the system in a single point attractor. In this case, positive virus replication detection increases (doubles) the number of attractor phenotypes (collectively represented by the mixed phenotypes nodes). A further unfolding of the respective paths to the leaves of the BDT involves decisions based on the presence of individual virus proteins (see Additional file 1 for details). All attractor phenotypes achieved that way display multi-stability.

The *variable stability analysis* tool provided in AEON allows to explore the individual nodes of the BDT for presence/absence of particular biological phenotypes (in our case represented as output variables in the BN). This helps us reveal the effect of input variables (virus proteins and drugs) on the studied biological phenotypes. In particular, the tool reports the number of instances exhibiting the given variable stable (True or False) or unstable in the selected node of the BDT. This allows us to characterise the effect of a given input variable on the number of instances presenting the studied phenotypes. In Table 1, we show the results for inputs with high information gain affecting the bifurcations. E.g., the presence of Nsp15 viral protein has no influence on the stability or bistability of interferon production and the presence of INFL. However, it has a negative effect on the presence of IIR in a single point attractor while increasing the number of instances where IIR becomes bistable. From this perspective, the effect of the drugs is interesting. In particular, GRL0617 contributes to increased IIR (in both stable and bistable regimes). Moreover, it does not affect the stable interferon production or stable INFL, and it increases the presence of bistable IIR and INFL. On the contrary, the effect of azithromycin is destabilising to all studied biological phenotypes. In the case of INFL and interferon production, this is the required feature. Anyway, it causes a potentially undesired side-effect for IIR.

**Table 1:** Qualitative influence of individual components on the stabilisation (more/less prominent or unchanged) of a particular phenotype (column) in either stable ($\odot$) or bistable ($\odot \odot$) regime

Component	Interf. production		IIR		INFL	
	${\mathbf{ \odot }}$	$\odot$ ${\mathbf{ \odot }}$	${\mathbf{ \odot }}$	${\mathbf{ \odot }}$ ${\mathbf{ \odot }}$	${\mathbf{ \odot }}$	${\mathbf{ \odot }}$ ${\mathbf{ \odot }}$
N	$\searrow$	$\searrow$	$\searrow$	$\nearrow$	$\searrow$	$\nearrow$
Nsp15	−	−	$\searrow$	$\nearrow$	−	−
GRL0617	−	$\nearrow$	$\nearrow$	$\nearrow$	−	$\nearrow$
azithromycin	$\searrow$	$\nearrow$	$\searrow$	$\nearrow$	$\searrow$	$\nearrow$

## Discussion

The Boolean abstraction of the interferon signalling model shows interesting insights into the non-linear dynamics driven by regulatory mechanisms of kinases, phosphatases, and other involved protein molecules (including virus proteins) reflected in the model. In contrast to the traditional quantitative models, e.g., represented as ODEs [61], it is not necessary to known the details of reaction kinetics to unfold the impact of regulatory feedback. This allows studying processes such as complex immune system networks that are not entirely known at the kinetic level [62, 63]. In particular, Boolean network models can predict the long-term dynamic patterns of a biological system under internal and environmental perturbations, and that way uncover the mechanisms behind biological phenotypes observed *in vivo* [10].

Our method creates a working analogy to the concept of bifurcation analysis widely used in kinetic modelling to reveal the impact of kinetic parameters on the system’s stability [64]. In our case, the bifurcation is understood as an effect of perturbing logical parameters (and/or input variables) of the Boolean model. Bifurcations are therefore understood as crucial decisions in the settings of perturbed components, affecting the long-term dynamics patterns. This kind of reasoning is enormously important in situations where different external (and/or internal) stimuli control the stabilisation of markers related to several specific biological phenotypes [65].

The presented case study investigates the combined effect of signal transduction and gene regulation on the immune response and thus represents a typical example of such a situation. The decision on which subset of biological phenotypes is achieved is processed based on several inputs: (i) input stimuli of immune system cells (extracellular interferons produced by other cells, internal/external mechanisms detecting the presence of the virus in the cell/tissue), (ii) proteins transcribed from the viral DNA, and (iii) drug molecules. The BDTs constructed by AEON have shown which inputs represent the most significant decision points causing switching between different attractors. Based on the computed information gain, the crucial role has been reported by the drug GRL0617. In a recent study [66], GRL0617 has been suggested as an efficient small molecule drug suppressing the replication of the virus through supporting the innate immune response phenotype. Our case study is in good agreement with those findings. In particular, our analyses show that regardless of the activity of the other inputs (including the virus proteins), GRL0617 increases the presence of innate immune response and the production of the type-1 interferons.

The important feature of our tool is its unique capability to work fast with large-scale networks with a relatively large extent of unknown information. In particular, this allows the user to work interactively with the models, making several variants reflecting the considered hypotheses. The largest analysed model had almost 100 variables and 18 input parameters (the size of the state space $\sim$ $1.3\times 10^{30}$), while the computations took at most 10 seconds on common hardware (Mac mini 2014, 2.8GHz Intel Core i5, 8GB RAM).

The employed BN model can be easily extended when new knowledge of the involved processes is obtained. This includes, e.g., the role of the Triggering Receptor Expressed on Myeloid cells-like 4 (TREML4) [67], which is purely incorporated in the currently known pathway reconstructions. Moreover, the new variants of the model can be quickly aligned with new virus mutations – these might affect the regulations controlled by virus proteins, including potentially new virus protein structures. Newly designed drug molecules can be incorporated within the model to explore their effect on the presence (and character) of the attractors underlying the targeted biological phenotypes.

## Conclusions

This article presents novel features of our unique method for fully automated bifurcation analysis of large Boolean networks with partially unknown information on regulatory mechanisms. The unknown knowledge is represented using uninterpreted functions that appear in logical formulae, specifying individual variables’ asynchronous updates.

We believe that attractor bifurcation computed by AEON will shift the current technology toward a comprehensive analysis of uncertainty in BNs. These advancements can then enhance other tools aimed at, for example, control or synthesis. In the future, we would also like to further enhance AEON with the ability to automatically connect the discovered attractors with a specification of biological phenotypes (based on their long-term behaviour and the stability properties of significant network variables).

## Supplementary Information

**Additional file 1**: Exploring attractor bifurcations in Boolean networks. A full technical description of the algorithms, including all necessary formal definitions, followed by a more detailed description of the models used in the case study, including a full report of the obtained results.

## Data Availability

AEON software is open source and available online at https://biodivine.fi.muni.cz/aeon/. Presented models, experiments, and up-to-date version of AEON are published as part of the supplementary data on Zenodo https://doi.org/10.5281/zenodo.5082165.

## Abbreviations

BN: Boolean network
BDD: Binary decision diagram
BDT: Bifurcation decision tree
BSCC: Bottom strongly connected component

## Footnotes

1 https://fairdomhub.org/models/713
