# Supplementary material for: Exploring attractor bifurcations in Boolean networks
Source: BMC Bioinformatics. 2022 May 11;23:173. doi: 10.1186/s12859-022-04708-9 (PMC9092939; doi:10.1186/s12859-022-04708-9)
Supplement: Supplementary file 1 — Additional file 1: Exploring attractor bifurcations in Boolean networks. A full technical description of the algorithms, including all necessary formal definitions, followed by a more detailed description of the models used in the case study, including a full report of the obtained results. [file 12859_2022_4708_MOESM1_ESM.pdf]

Supplementary Material: Exploring Attractor Bifurcations in Boolean Networks

1 Algorithms and Methods

In this section, we cover the symbolic algorithms which enable bifurcation analysis in AEON. The material is based on the previous work published in [1, 2, 4], but also provides additional novel technical details. Furthermore, it serves as additional clarification of several technical aspects presented in the main manuscript.

1.1 Symbolic encoding

First, let us formally define Boolean networks and their dynamics, including parametrisations and uninterpreted functions. Subsequently, we discuss how this formalism is encoded symbolically in terms of binary decision diagrams.

1.1.1 Boolean networks

Definition 1. *A* Boolean network *is a tuple* $(\mathcal{V}, \mathcal{R}, \mathcal{F})$*, such that:*

- $\mathcal{V}$ *is the set of Boolean variables (denoted* $\mathtt{A}, \mathtt{B}, \ldots$*);*
- $\mathcal{R} \subseteq \mathcal{V} \times \mathcal{V}$ *is the set of regulations;*
- $\mathcal{F} = \{F_{\mathtt{A}} \mid \mathtt{A} \in \mathcal{V}\}$ *is a family of Boolean update functions.*

The signature of each update function $F_{\mathtt{A}}$ *is given by its regulatory context* $\mathcal{C}(\mathtt{A}) = \{\mathtt{B} \in \mathcal{V} \mid (\mathtt{B}, \mathtt{A}) \in \mathcal{R}\}$*. That is,* $F_{\mathtt{A}} : \{0,1\}^{\mathcal{C}(\mathtt{A})} \to \{0,1\}$.

Here, the members of $\mathcal{C}(\mathtt{A})$ are the *regulators* of $\mathtt{A}$, while $\mathtt{A}$ is the *target* of the regulations. Subsequently, the set of all possible Boolean valuations of network variables (i.e. $\{0,1\}^{\mathcal{V}}$) is the *state space* of the Boolean network.

For conciseness, given a state $s \in \{0,1\}^{\mathcal{V}}$, we abuse the notation slightly and write $F_{\mathtt{A}}(s)$ to denote the application of $F_{\mathtt{A}}$ to the state s restricted to the variables within the regulatory context $\mathcal{C}(\mathtt{A})$. Additionally, we write $s[\mathtt{A} \mapsto b]$ for $b \in \{0,1\}$ to denote a copy of s with the value of $\mathtt{A}$ fixed to b.

Asynchronous dynamics The dynamics of a Boolean network are described through its asynchronous state-transition graph.

Definition 2. *An* asynchronous state-transition graph *of a Boolean network* $(\mathcal{V}, \mathcal{R}, \mathcal{F})$ *is a pair* (V, E)*. Here,* V *are the states of the Boolean network* $V = \{0,1\}^{\mathcal{V}}$*. The edge relation* $E \subseteq V \times V$ *is then constructed as follows:*

$$(u, v) \in E \Leftrightarrow u \neq v \wedge \exists \mathtt{A} \in \mathcal{V}.\ v = u[\mathtt{A} \mapsto F_{\mathtt{A}}(u)]$$

Intuitively, an edge is created between two states which differ in exactly one variable, such that this variable is updated using its associated update function. We write $u \to v$ when $(u, v) \in E$, and $u \to^* v$ when $(u, v) \in E^*$ (the reflexive and transitive closure of E).

Within the state transition graph, we recognise *attractors* as important behavioural features of the underlying Boolean network:

Definition 3. *An* attractor *is the bottom SCC of the state-transition graph of a Boolean network* $(\mathcal{V}, \mathcal{R}, \mathcal{F})$*. That is, a maximal set* $A \subseteq \{0,1\}^{\mathcal{V}}$ *such that for every two* $u, v \in A$*, we have* $u \to^* v$ *and* $v \to^* u$*, but at the same time, there is no* $w \in V \setminus A$ *s.t.* $u \to w$*.*

We generally recognise three types of attractors:

- Set A is a *stable* attractor when it consists of a single state.
- Set A is an *oscillating* attractor when its state-transition graph is a cycle.
- Set A is a *disordered* attractor when it is neither stable nor oscillating.

Later, we discuss how attractors can be efficiently detected in the state-transition graph of Boolean network.

1.1.2 Parametrised Boolean networks

To express possible uncertainty in the behaviour of a Boolean network, we define the notion of *parametrised* Boolean networks:

Definition 4. *A* parametrised Boolean network *is a tuple* $(\mathcal{V}, \mathcal{R}, \mathcal{P}, \widehat{\mathcal{F}})$*. Here,* $\mathcal{V}$ *and* $\mathcal{R}$ *have the same purpose as in Definition 1. Additionally,* $\mathcal{P}$ *is the set of* logical parameters*, and* $\widehat{\mathcal{F}} = \{\widehat{F}_{\mathtt{A}} \mid \mathtt{A} \in \mathcal{V}\}$ *the set of* parametrised *update functions.*

The signature of each parametrised update function is determined by its regulatory context and *the set of logical parameters:* $\widehat{F}_{\mathtt{A}} : \{0,1\}^{\mathcal{C}(\mathtt{A}) \cup \mathcal{P}}$.

Intuitively, logical parameters represent unknown constant values which influence the behaviour of the network, but do not change during its evolution. Consequently, the behaviour of many different Boolean network can be described using a single parametrised network.

Similar to the network's state space, we define the network's parameter space to be the set $\{0,1\}^{\mathcal{P}}$ and we denote the members of this set as *parametrisations*.

For a state $s \in \{0,1\}^{\mathcal{V}}$ and a parametrisation $p \in \{0,1\}^{\mathcal{P}}$, we write $\widehat{F}_{\mathtt{A}}(s \cup p)$ to denote the application of $\widehat{F}_{\mathtt{A}}$ to the joint valuation of network variables and parametrisations.

Note that in practice, we may sometimes only operate within a subset of biologically relevant parametrisations $Valid \subseteq \{0,1\}^{\mathcal{P}}$. Nevertheless, this is easily captured within the algorithms in this document by substituting the set of all parametrisations $\{0,1\}^{\mathcal{P}}$ for the set $Valid$ (complement operation $\overline{A}$ is handled using $Valid \setminus A$).

Asynchronous dynamics To compactly describe the asynchronous dynamics of such a network, we rely on a *coloured* state-transition graph. In such a graph, a set of colours is used to distinguish multiple possible edge relations over the same set of vertices:

Definition 5. *An* asynchronous state-transition graph *of a fixed* parametrised *Boolean network $(\mathcal{V}, \mathcal{R}, \mathcal{P}, \mathcal{F})$ is a tuple (V, C, E). Here, V (vertices) are the states of the Boolean network, $V = \{0,1\}^{\mathcal{V}}$, and C (colours) are the parametrisations, $C = \{0,1\}^{\mathcal{P}}$. The edge relation of the graph, $E \subseteq V \times C \times V$, is then constructed as follows:*

$$(u, c, v) \in E \Leftrightarrow u \neq v \wedge \exists \mathtt{A} \in \mathcal{V}.\ v = u[\mathtt{A} \mapsto \widehat{F}_{\mathtt{A}}(u \cup c)]$$

In other words, the coloured state-transition graph collectively describes the state-transition graphs of every parametrisation of the associated network. For the rest of the text, we thus consider colours and parametrisations to be interchangeable.

Note that the notion of attractors does not transfer naturally to the domain of coloured graphs, as each colour can induce a completely different set of attractors. Nevertheless, we can write that the set A is an attractor *in colour c* if it is an attractor in the state-transition graph which arises after fixing the colour c.

1.1.3 Uninterpreted Boolean functions

Logical parameters allow us to richly capture uncertainty in the update functions of a Boolean network, but are not always the most intuitive tool for this task. In many cases, when a larger part of the network's dynamics is unknown, such uncertainty is better captured through uninterpreted Boolean functions.

To this end, AEON supports the usage of uninterpreted Boolean functions within the definition of update functions in parametrised Boolean networks. These uninterpreted functions are then automatically translated to logical parameters within the parametrised network. The dynamics of such a network then still correspond to our description above.

Formally, we assume a finite collection of uninterpreted Boolean functions $\mathtt{K}^{(a_{\mathtt{K}})}, \mathtt{L}^{(a_{\mathtt{L}})} \ldots$, where the value $a_{\mathtt{X}}$ is the arity of the function and can be omitted when clear from context. Our goal is to allow the usage of such Boolean functions within the declarations of update function within our parametrised network.

Now, notice that an uninterpreted Boolean function of arity zero is equivalent to a logical parameter. Consequently, any Boolean expression which only uses uninterpreted functions of arity zero can be used to define a parametrised update function, assuming the network's logical parameters include these zero-arity uninterpreted function symbols.

Using the following expansion rule, we can recursively transform an expression with uninterpreted Boolean functions of arbitrary arity a into an expression which only uses zero-arity uninterpreted functions.

$$\mathtt{K}^{(a)}(\alpha_1, \dots, \alpha_a) \equiv (\alpha_1 \Rightarrow \mathtt{K}_1^{(a-1)}(\alpha_2, \dots, \alpha_a)) \wedge (\neg\alpha_1 \Rightarrow \mathtt{K}_2^{(a-1)}(\alpha_2, \dots, \alpha_a))$$

Here, α_i are arbitrary Boolean expressions (possibly including other uninterpreted Boolean functions). Furthermore, $\mathtt{K}_1$ and $\mathtt{K}_2$ are two fresh uninterpreted Boolean functions specific to $\mathtt{K}$. That is, every occurrence of $\mathtt{K}$ is expanded using these two functions, but they are not used anywhere else within the model.

By recursively applying this expansion, an uninterpreted function $\mathtt{K}^{(a)}$ is replaced with 2^a zero-arity uninterpreted functions equivalent to logical parameters. These logical parameters then correspond exactly to the individual rows in the truth table of function $\mathtt{K}$. As such, the resulting network has exponentially many logical parameters with respect to the arity of the uninterpreted functions used to define the network.

In the following, we generally assume that the input of the method is a parametrised Boolean network; however, this network can be given using expressions which contain uninterpreted Boolean functions. These expressions are then automatically translated using the aforementioned expansion rule into logical parameters.

1.1.4 Symbolic Representation of BNs

As a symbolic representation, a natural choice are Reduced Ordered Binary Decision Diagrams (ROBDD, or simply BDD) [5], which can concisely encode Boolean functions or relations of Boolean vectors. Specifically, in AEON [3], we use a specialised internal implementation written in Rust.

Our goal is to symbolically represent and manipulated the coloured state-transition graph of a parametrised Boolean network. Recall that this graph consists of vertices $V = \{0,1\}^{\mathcal{V}}$, colours $C = \{0,1\}^{\mathcal{P}}$ and a coloured edge relation $E \subseteq V \times C \times V$.

Since a Boolean network consists of $|\mathcal{V}|$ Boolean variables and $|\mathcal{P}|$ Boolean inputs, any subset of V, C, or a relation $X \subseteq V \times C$ (a *coloured set of vertices*) can be seen as a Boolean formula (also denoted X) over the network variables and logical parameters. That is, each network variable and logical parameter corresponds to one decision variable of the BDD. Here, a pair (s, c) belongs to such a relation iff it represents a satisfying assignment of this formula X. For relations of higher arity, fresh decision variables are created for each component of the relation. Standard set operations then correspond to logical operations on such formulae ($\wedge \equiv \cap$, $\vee \equiv \cup$, etc.).

Relation operations are similarly implementable using BDD primitives. In particular, existential quantification of a single decision variable (e.g. $\exists s_i.X$

or $\exists c_j.X$) is a native operation on BDDs. Consequently, colour-projection on relations (see below) can be simply implemented using quantification over all network variables.

$$\textsc{Colours}(X \subseteq V \times C) = \{c \in C \mid \exists v \in V.\ (v, c) \in X\}$$

To encode the network dynamics, notice that every update function $\widehat{F}_{\mathtt{A}}$ can be directly represented as a separate BDD. From such BDDs, we can build one large BDD describing the whole coloured transition relation, which is traditionally used for the computation of operations $\textsc{Pre}$ (set of predecessor vertices: $\textsc{Pre}(X \subseteq V \times C) = \{(u, c) \mid \exists v \in V.\ (u, c, v) \in E \wedge (v, c) \in X\}$) and $\textsc{Post}$ (set of successor vertices: $\textsc{Post}(X \subseteq V \times C) = \{(v, c) \mid \exists u \in V.\ (u, c, v) \in E \wedge (u, c) \in X\}$). But the symbolic representation of such relation is often prohibitively complex for asynchronous systems. Instead, we compute $\textsc{Pre}$ and $\textsc{Post}$ using partial results for individual variables, which uses more symbolic operations but is less likely to cause a blow-up in the size of the BDD:

$$\begin{aligned}
\textsc{VarPost}(\mathtt{A} \in \mathcal{V}, X \subseteq V \times C) &= (X \wedge (\widehat{F}_{\mathtt{A}} \not\Leftrightarrow \mathtt{A}))[\mathtt{A} \mapsto \neg\mathtt{A}] \\
\textsc{VarPre}(\mathtt{A} \in \mathcal{V}, X \subseteq V \times C) &= X[\mathtt{A} \mapsto \neg\mathtt{A}] \wedge (\widehat{F}_{\mathtt{A}} \not\Leftrightarrow \mathtt{A}) \\
\textsc{Post}(X) &= \bigvee_{\mathtt{A} \in \mathcal{V}} \textsc{VarPost}(\mathtt{A}, X) \\
\textsc{Pre}(X) &= \bigvee_{\mathtt{A} \in \mathcal{V}} \textsc{VarPre}(\mathtt{A}, X)
\end{aligned}$$

Here, $[\mathtt{A} \mapsto \neg\mathtt{A}]$ is the standard substitution operation, which we use to flip the value of variable $\mathtt{A}$ in the resulting formula if it does not agree with the output of $\widehat{F}_{\mathtt{A}}$. Note that this operation can be also implemented directly on the structure of the BDD by exchanging the children of decision nodes conditioning on $\mathtt{A}$. Also note that sub-formulae that do not depend on X can be pre-computed once for the whole run of the algorithm.

In the following, we thus assume a parametrised Boolean network (possibly defined using uninterpreted Boolean functions) using the aforementioned symbolic representation and the corresponding operations.

1.2 Attractor detection

In this section, we describe the attractor detection techniques used by AEON. This part is primarily adapted from [4]. Note that in [4], the technique is presented on standard state-transition graphs. Here, we present it with modifications that apply it to coloured graphs arising from parametrised BNs.

1.2.1 Basic Symbolic BSCC Detection

First, we discuss a BSCC detection algorithm from [7], which is a well known core of our method. The method is summarised in Algorithm 1, which shows the main procedure (BSCC) as well as the reachability procedures $\textsc{Bwd}$ and

```
Function BSCC(universe ⊆ V × C)
  while universe ≠ ∅ do
    pivot ← PickVertex(universe);
    basin ← Bwd*({pivot}, universe);
    forward ← {pivot};
    repeat
      (fixpoint, forward) ← Fwd(forward, universe);
    until fixpoint or forward ⊈ basin;
    if forward ⊆ basin then
      report forward as attractor;
    end
    universe ← universe \ basin;
  end
Function Bwd*(reachable ⊆ V × C, universe ⊆ V × C)
  repeat
    (fixpoint, reachable) ← Bwd(reachable, universe);
  until fixpoint;
  return reachable;
Function Bwd(reachable ⊆ V × C, universe ⊆ V × C)
  for A ∈ 𝒱 do
    pre ← universe ∩ VarPre(A, reachable);
    if pre ⊈ reachable then
      return (false, reachable ∪ pre);
    end
  end
  return (true, reachable);
```

Algorithm 1: Basic BSCC detection algorithm with saturation.

BWD*, which we also use in the later sections. We omit the pseudocode for FWD and FWD*, as they are identical to the BWD case, only swapping VARPRE for VARPOST.

Reachability and Saturation The forward and backward reachability procedures are divided into two methods each, FWD, BWD, FWD* and BWD*. Since they are functionally symmetrical, we only explicitly discuss backward reachability, with everything directly translating to forward reachability as well.

BWD performs a single backward reachability step and returns the new set of states together with an indication of whether a fixed point has been reached (i.e. whether no new states have been discovered). Note that in classical saturation, once BWD selects transition under `A`, it is typically applied repeatedly. However, in Boolean networks, multiple subsequent applications of a single transition would not yield any benefit.

BWD* then simply wraps BWD into a cycle that actually computes the full fixed point of the `reachable` set. This separation into two sub-procedures allows us to perform reachability step-by-step or even interleave multiple reachability

procedures. Remember that for saturation to work well, the ordering of labels needs to follow the ordering of variables in the symbolic representation.

Xie-Beerel Algorithm The main algorithm relies on the well-known observation that for a fixed `pivot` vertex, the SCC of this vertex can be computed as the intersection of vertices forward and backward reachable from `pivot`. When searching for BSCCs, we can easily extend this with two extra observations: First, `pivot` is in a BSCC when only the SCC itself is forward-reachable from `pivot`. Second, a vertex backward-reachable from `pivot` is either in the same SCC as `pivot` (in which case it is in a BSCC iff `pivot` is in a BSCC), or it is not in a BSCC.

Based on these two extra observations, the original algorithm is modified in two ways: First, not just the SCC around `pivot`, but all backward-reachable vertices are eliminated at the end of each iteration. Second, the backward reachability from `pivot` is computed in full, as these are the vertices we can eliminate. However, the forward reachability is terminated early if it leaves the backward-reachable set, since this implies that `pivot` does not belong to a BSCC.

Now, for a coloured graph, this reasoning has to be slightly modified, since the attractors of such a graph can be different for different colours. As the algorithm progresses, there may be no single vertex that can be chosen as `pivot` which would cover ever colour still present in `universe`. Consequently, we define a symbolic operation PickVertex which produces a coloured set of vertices X (i.e. $X \subseteq V \times C$) such that for every colour in `universe`, X contains exactly one pivot vertex. This operation can be relatively easily implemented using standard logical operations on BDDs.

In [7], the authors show very impressive performance numbers for this simple algorithm. However, there are two drawbacks, which we believe can be improved significantly. And as we demonstrate in the evaluation, while powerful, this algorithm certainly has limits on some real-world models.

First, the performance of this method is directly tied to the selection of the `pivot` vertex. If the BSCCs of the graph are relatively small, the probability of picking a right pivot is also tiny (remember, even an SCC with 2^{100} vertices is only a minuscule fraction of a graph with 2^{1000} vertices). As a consequence, the algorithm may require a lot of pivots to explore the entire graph. Second, the overall complexity is limited by the diameter of the whole graph instead of the diameter of the BSCCs. Even if the `pivot` is picked perfectly, the algorithm still has to explore each BSCC's whole basin sequentially. To some extent, this is inevitable; however, as we hope to demonstrate in the next section, it is not always necessary.

To sum up, Algorithm 1 is a powerful tool for the detection of BSCCs. However, it performs best in graphs where the BSCCs either form a large portion of the state space or have basins of small diameter, allowing the algorithm to converge quickly.

```
Function Reduce(pivots ⊆ V × C, universe ⊆ V × C)
    forward ← Fwd*(pivots, universe);
    extendedComponent ← Bwd*(pivots, forward);
    bottom ← forward \ extendedComponent;
    if universe ≠ forward then
        basin ← Bwd*(forward, universe);
        universe ← universe \ (basin \ forward);
    end
    if bottom ≠ ∅ then
        basin ← Bwd*(bottom, universe);
        universe ← universe \ (basin \ bottom);
    end
    return universe;
```

Algorithm 2: Core reduction principle

1.2.2 Transition guided reduction

In this section, we introduce a technique that we call *transition guided reduction* (TGR) to eliminate a large portion of non-BSCC states. Algorithm 1 can then perform much better on this reduced state space.

We present the technique in two steps: First, in Algorithm 2, we present the core principle of the reduction procedure and prove its correctness. This approach is generally applicable to any directed graph. Then in Algorithm 3 we show how to apply Algorithm 2 in the context of a labelled transition system. Here, we can exploit the knowledge of the transition labels to guide the reduction.

The reduction principle is described in Algorithm 2. Given a coloured set of `pivot` states and the current `universe` of all considered states, the method starts by computing `forward` — the set of all states reachable from the `pivot` states. Using this `forward` set, we then compute the `extendedComponent` of the given `pivot` states. Formally, an extended component of set X is a subset $X' \subseteq S$ that contains all states from X, as well as all paths between the states in X.

We can observe the following properties:

- The `forward` set is SCC-closed (every SCC is either fully contained in the set or in its complement), as it is the result of a reachability procedure. Thus any state that can reach but is not contained in `forward` is not a part of any BSCC.
- The set `bottom` (i.e. `forward` \ `extendedComponent`) is also SCC-closed (as it is the difference of two SCC-closed sets). Notice that if this set is not empty, it must contain at least one BSCC, and also that any state that can reach `bottom` but is not contained in it is necessarily not a part of any BSCC.

The algorithm then computes the two sets of states that definitely do not contain a BSCC according to these observations and discards these sets from the state space. This is done on lines 2–2 and 2–2, respectively.

Now we can formulate the following observation (formal proof available in [4]):

Claim 1. *If pair* $(s, c) \in$ `universe` *is discarded by Algorithm 2, then vertex s it is not part of any BSCC in colour c.*

Function TGR(`universe` $\subseteq V \times C$)
  for `A` $\in \mathcal{V}$ **do**
    `universe` $\leftarrow$ REDUCE(CANPOST(`A`, `universe`), `universe`);
  end
  return `universe`;

Algorithm 3: Transition Guided Reduction

However, this does not provide any guidance as to which `pivots` should we select for the reduction or why. This is addressed in Algorithm 3. Here, we go through all the available variables `A` $\in \mathcal{V}$ and select as the `pivots` the set of all the states that can fire a transition under `A`. Here, CANPOST is the subset of `universe` for which a successor exists using the VARPOST method.

To intuitively understand why this method is effective, we present the following claims (again, proven within [4]):

Claim 2. *Given a* trivial *BSCC (stable attractor), the whole basin of this SCC is discarded by Algorithm 3.*

Claim 3. *If a pair* (s, c) *is not discarded by Algorithm 3, then all paths (under colours c) starting in s in the reduced state space only modify the same variables as the ones contained in the BSCCs reachable from s.*

The first claim highlights an important property of the reduction: if variable is constant in an attractor, all states in its basin where this variable is modified will be eliminated. Real-world network rarely modify all variables within attractors. Thus by using this pre-processing step, we can greatly simplify the work of Algorithm 1 by pruning “easily identifiable” non-BSCC states.

The effectiveness of this reduction can be further improved by employing interleaving (see [4]) instead of the for cycle within Algorithm 3.

1.3 Behaviour classification

Our ultimate goal is to produce and study the attractor bifurcation function $\mathcal{A} \to \mathfrak{C}$, where $\mathfrak{C}$ are the behaviour classes induced by some attractor classification. So far, we have presented an algorithm for attractor detection in parametrised Boolean networks. This algorithm produces coloured sets of vertices, such that every attractor for every colour is eventually guaranteed to appear in one of these sets.

Note that classification of attractors based on stability, oscillation and disorder is trivial. For stability, we simply need to detect colours where the attractor set is a singleton. For oscillation, we need to detect cases where all the vertices have only a single successor in the state-transition graph. Practically, we consider this classification in our tool. Nevertheless, this section presents a general method for constructing the bifurcation function for various classifiers. This is unpublished material.

Let us assume that we have a fixed parameter space (set of colours) $C = \{0,1\}^{\mathcal{P}}$. Additionally, let us assume a set of *behavioural classes* $\mathfrak{C}$, and a *feature detection algorithm* which can *report* on the fly that a system under consideration (such as a Boolean network) exhibits an *atomic behavioural feature* $b \in \mathfrak{B} \subseteq \mathfrak{C}$ for a specific subset of parametrisations.

As a simple example, assume that we only want to *count* the number of attractors for each colour. Then, $\mathfrak{C} = \mathbb{N}_0$, and $\mathfrak{B} = \{1\}$. That is, a behavioural class is the absolute count of attractors, and an atomic feature is the existence of a single attractor. An attractor detection algorithm then reports that a new attractor (a *feature* $b = 1$) has been discovered for a particular set of network parametrisations $Q \subseteq C$ (the algorithm also reports attractor states, but we can disregard these for the purposes of our example).

We require that $\mathfrak{C}$ is a commutative monoid with a zero element (denoted ϵ) equipped with an operation $\oplus$. The behavioural class c_p for every parametrisation $p \in C$ is then given as the sum of all atomic features reported for this p by our feature detection algorithm, i.e. $c_p = b_1 \oplus \ldots \oplus b_k$ for some k.

Continuing our example of attractor counting, the operation $\oplus$ is addition, and for a parametrisation p that admits five attractors, the detection algorithm will report five atomic features (five ones) which together give $c_p = 5$.

Naturally, $\mathfrak{C}$ can also have a richer structure: for example, our practical application of detecting stability, oscillation and disorder results in $\mathfrak{C} = \mathbb{N}_0^3$.

Finally, let us note that since $\mathfrak{C}$ is a commutative monoid, it also has an associated preorder where $a \leq c$ when $a + b = c$ for some b. For the purposes of our classifier, we strengthen this observation and require that $\mathfrak{C}$ is in fact at least a partial order (i.e., we cannot have $a \leq b$ and $b \leq a$ without $a = b$).

To incrementally build our classification map, we rely on the procedure UpdateClassification defined in Algorithm 4. Here, we start with a $\mathcal{A}$ which initially assigns all parametrisations $p \in C$ to the zero element of $\mathfrak{C}$. Then, as behavioural features are discovered, they are reported using the procedure UpdateClassification(b, Q). Now, we use the fact that $\mathfrak{C}$ has a partial order, and thus we can iterate through the keys of the $\mathcal{A}$ (the encountered behavioural classes) in a decreasing order. Of course, since this is only a partial order, there may be incomparable pairs in such set, but we only require that whenever $a \leq b$, a is encountered *after* b by the loop on Line 4 (i.e. incomparable elements can be considered in any order). Then, during each iteration, we determine the set of parametrisations (the set *Update*) associated with $c \in$ Keys$(\mathcal{A})$ whose class needs to be updated. If this set is not empty, it is transferred to a new class.

To understand why this procedure is correct, note that the loop on Line 4 maintains an invariant that $\mathcal{A}$ partitions the set C between classes in $\mathfrak{C}$. That

$\mathcal{A} \leftarrow \{\epsilon \mapsto P\}$;
Function UPDATECLASSIFICATION($b \in \mathfrak{B}, Q \subseteq P$)
  for c **in decreasing** KEYS($\mathcal{A}$) **do**
    $\mathit{Update} \leftarrow Q \cap \mathcal{A}(c)$;
    if $\mathit{Update} \neq \emptyset$ **then**
      $\mathcal{A}(c) \leftarrow \mathcal{A}(c) \setminus \mathit{Update}$;
      $\mathcal{A}(b \oplus c) \leftarrow \mathcal{A}(b \oplus c) \cup \mathit{Update}$;
    end
  end

Algorithm 4: Incremental symbolic classification

is, $C = \bigcup_{c \in \mathfrak{C}} \mathcal{A}(c)$, and for any two $a, b \in \mathfrak{C}$, we have that $\mathcal{A}(a) \cap \mathcal{A}(b)$ is empty. This is easy to see, since initially, we have $\mathcal{A}(\epsilon) = P$ (and for every other $c \in \mathfrak{C}$, $\mathcal{A}(c) = \emptyset$). Subsequently, in every iteration, we only transfer the *Update* set between two classes. We thus cannot remove a parametrisation, nor can we associate it simultaneously with two different classes.

The order of iteration then ensures that parametrisations are actually associated with the correct classes. Consider a scenario where a set Update_{c_1} is transferred from class c_1 to $c_1 \oplus b = c_2$ in one iteration. Now assume that c_2 is encountered by loop on Line 4 *after* c_1. Clearly, Update_{c_2} will be non-empty and a superset of Update_{c_1}. Consequently, Update_{c_1} is moved again, this time into $c_2 \oplus b = c_1 \oplus b \oplus b$. Intuitively, b is "counted twice" in this scenario.

However, due to the fact that the algorithm explores classes in a decreasing partial order, this situation cannot happen. We have that $b \oplus c \geq c$, hence $b \oplus c$ is always processed before c (or, in a special case where $b = \epsilon$, they are processed in a single iteration, but here, *ClassMap* is not modified). Consequently, every parametrisation is moved at most once during any invocation of UPDATECLASSIFICATION.

1.4 Bifurcation decision tree inference

Once we obtain a $\mathcal{A}$ as outlined in the previous section, a natural goal would be to somehow visualise this map. It may not be possible to produce a readable table or a plot which would describe the $\mathcal{A}$ visually. For this reason, we propose usage of decision trees as a reasonably universal visualisation tool which addresses this issue.

This leaves us with two problems: First, there are many different decision trees that we can generate from a single $\mathcal{A}$. In general, we cannot efficiently determine which decision will lead to a more compact tree, however, we can still employ heuristics that address this problem. Second, the sets of parametrisations in $\mathcal{A}$ are symbolically encoded. And while the *bifurcation decision tree* (BDT) will have to be explicit (so that we can draw it), it still needs to be generated using these symbolic sets, as instantiating them explicitly could easily exceed the available computer memory.

Consequently, to provide an automated procedure for generation of bifurcation decision trees, we take the heuristic algorithm known as ID3 [6], and adapt it to symbolic datasets, such that it can be used to process our $\mathcal{A}$. This approach has been previously explored within [2].

Function $\textsc{LearnTree}(B_{c_1}, \ldots, B_{c_k})$
  if $k = 1$ **then return** $\textsc{LeafNode}(c_1)$;
  $(c_{max}, r_{max}) = \textsc{Majority}(B_{c_1}, \ldots, B_{c_k})$;
  if $r_{max} \geq$ `precision` **then return** $\textsc{LeafNode}(c_{max})$;
  `entropy` $\leftarrow \textsc{Entropy}(B_{c_1}, \ldots, B_{c_k})$;
  for $A \in \mathfrak{A}$ **do**
    `positive` $\leftarrow \textsc{Entropy}(\forall i.\ B_{c_i} \cap A)$;
    `negative` $\leftarrow \textsc{Entropy}(\forall i.\ B_{c_i} \cap \overline{A})$;
    $\texttt{gain}_A \leftarrow \texttt{entropy} - (\frac{1}{2}\texttt{positive} + \frac{1}{2}\texttt{negative})$;
  end
  $D \leftarrow A \in \mathfrak{A}$ with maximal $\texttt{gain}_A$;
  `r_node` $\leftarrow \textsc{LearnTree}(\forall i.\ B_{c_i} \cap D)$;
  `l_node` $\leftarrow \textsc{LearnTree}(\forall i.\ B_{c_i} \cap \overline{D})$;
  return $\textsc{DecisionNode}(D, \texttt{l\_node}, \texttt{r\_node})$;
Function $\textsc{Entropy}(B_1, \ldots, B_k)$
  $B_{all} \leftarrow \cup_{i=1}^{k} B_i$;
  return $\sum_{i=1}^{k} -\frac{|B_i|}{|B_{all}|} log_2(\frac{|B_i|}{|B_{all}|})$;
Function $\textsc{Majority}(B_{c_1}, \ldots, B_{c_k})$
  $B_{all} \leftarrow \cup_{i=1}^{k} B_{c_i}$;
  $\forall i.\ r_i \leftarrow \frac{|B_{c_i}|}{|B_{all}|}$;
  return (c_i, r_i) **with maximal** r_i;

Algorithm 5: Symbolic ID3

This adaptation is presented in Algorithm 5. Here, we assume that $\mathcal{A}$ is expanded into a collection of symbolic sets $B_{c_1}, \ldots, B_{c_k}$, where $c_1, \ldots, c_k$ are the keys (i.e. the behaviour classes) used within $\mathcal{A}$, and each set B_{c_i} contains the parametrisations assigned to this class (i.e. $\mathcal{A}(c_i) = B_{c_i}$). Furthermore, we assume there is a collection of *decision attributes* $\mathfrak{A}$, such that each $A \in \mathfrak{A}$ is a symbolically represented subset of P (our parameter space). In this representation, A can be seen as a binary decision attribute where a parametrisation $p \in C$ either satisfies it ($p \in A$), or not ($p \in \overline{A}$).

For simplicity, let us assume that the decision attributes represent the validity of individual logical parameters of a Boolean network. That is, $\mathfrak{A} = \{ A_{\texttt{P}} \mid \texttt{P} \in \mathcal{P} \}$ where $A_{\texttt{P}} = \{ x \in \{0,1\}^{\mathcal{P}} \mid x(\texttt{P}) = 1 \}$. However, for other applications, we may also choose different, more complicated decision attributes. In theory, our method can support any attributes that can be encoded into a symbolic set.

Algorithm description Now, the algorithm itself works as follows: For a given dataset, the algorithm considers all available decision attributes, and

selects the one with the highest information gain. Here, the information gain is computed as a difference in information entropy before and after conditioning on the decision attribute.

Given a symbolic dataset specified as a collection of BDDs, ENTROPY in Algorithm 5 computes the overall information entropy of the dataset. Note that we use $|B|$ to denote the cardinality of the set B – this information is easily computed using dynamic programming while traversing the graph of the corresponding BDD.

The procedure LEARNTREE then conditions on individual decision attributes $A \in \mathfrak{A}$ by intersecting B_{c_i} with A or $\overline{A}$ (attributes where one of the intersections is empty for all B_{c_i} are automatically discarded). Based on these values, it computes the information gain $\texttt{gain}_A$ for each attribute and selects the decision attribute D with the highest information gain. Finally, a decision node is created and the remaining datasets are processed recursively.

A leaf node is created by LEARNTREE once a single class remains. Alternatively, we can also specify a desired `precision` constant threshold which is applied to the whole tree. Then, we create a leaf node whenever the proportion of a single class in the whole dataset is higher than this threshold. For example, if we specify a precision of 0.95, a leaf node is created if 95% of parametrisations belong to a single class. While this produces an inexact tree, it can also significantly reduce its size while preserving reasonable amount of information with a clearly bounded error.

Overall, this algorithm allows us to easily process even very large sets of parametrisations that can only be represented symbolically. However, note that the algorithm still has to explicitly iterate through the list of the possible decision attributes for each created decision node, which can be substantial. As future work, similar to the incremental classification approach, one could explore whether some of these calculations could be also efficiently implemented using multi-valued decision diagrams.

1.5 Conclusions

Overall, this section presented the algorithmic techniques necessary to enable bifurcation analysis of parametrised Boolean networks in AEON. This includes translation of uninterpreted Boolean functions into logical parameters; symbolic encoding of the asynchronous state-transition graph (for parametrised BNs); attractor detection through transition guided reduction (originally presented for monochromatic state-transition graphs); classification of the network's behaviour classes into a symbolically represented bifurcation function; and a symbolic learning technique for automated inference of bifurcation decision trees.

2 AEON

This supplement contains a snapshot of the version of the tool AEON used to perform the experiments in the paper. However, the tool is also available

online at `https://biodivine.fi.muni.cz/aeon/` and we recommend you use the online up-to-date version when possible.

The bundled AEON snapshot contains:

- `aeon/manual`: A comprehensive manual describing AEON's functionality.
- `aeon/web` and `aeon/source`: The source code of the web-based GUI and native compute engine used by AEON.
- `aeon/bin`: Pre-compiled binaries of the AEON compute engine for the three major PC operating systems.

Further guidance about using these resources is given in `aeon/README.md`.

3 Original Model

The studied interferon model was originally constructed based on the SBGN curated pathway available at `https://fairdomhub.org/models/713`. The actual `.sbml` model was then obtained from the COVID-19 disease map project at `https://git-r3lab.uni.lu/covid/models`. The model is available as both `model/original.sbml` and `model/original.aeon` file in this supplement. Note that attached model uses a different layout of the regulatory graph than in the original `.sbml` to ease readability. The regulatory graph of the model is shown in Figure 1. The full resolution figure is also available in the file `figures/model_original_full.png` and can be reproduced in AEON by opening either the `.sbml` or `.aeon` model file. Also, note that AEON does not support special characters in variable names (aside from underscore), so all variable names have been normalized to be compatible with AEON.

A table summarising the bifurcation function for this original model (as computed by AEON) is shown in Figure 2. To reproduce this figure, run bifurcation analysis (*Start Analysis* button) in AEON's interface.

As we can see, this bifurcation function contains a (relatively) high number of single state attractors that appear simultaneously (reporting multi-stability). However, by inspecting the individual attractors (using the *Attractor* button in AEON's interface), we can actually uncover that most of these multi-stabilities do not alter the network phenotypes (**`Immune_response_phenotype`** and **`Inflammation_phenotype`** variables). In order to make the bifurcation function easier to read, we thus provide and analyse a reduced model that restricts the number of possible multi-stable attractors.

4 Reduced Model

The original model contains a complex of three mutually dependent variables, `NFKBIA`, `NFKB-NFKBIA-complex` and `NFKB1-cell`. This complex is visible in the upper-middle section of the regulatory graph of the original model (we recommend opening the model in AEON to see a full zoomable version). By inspecting

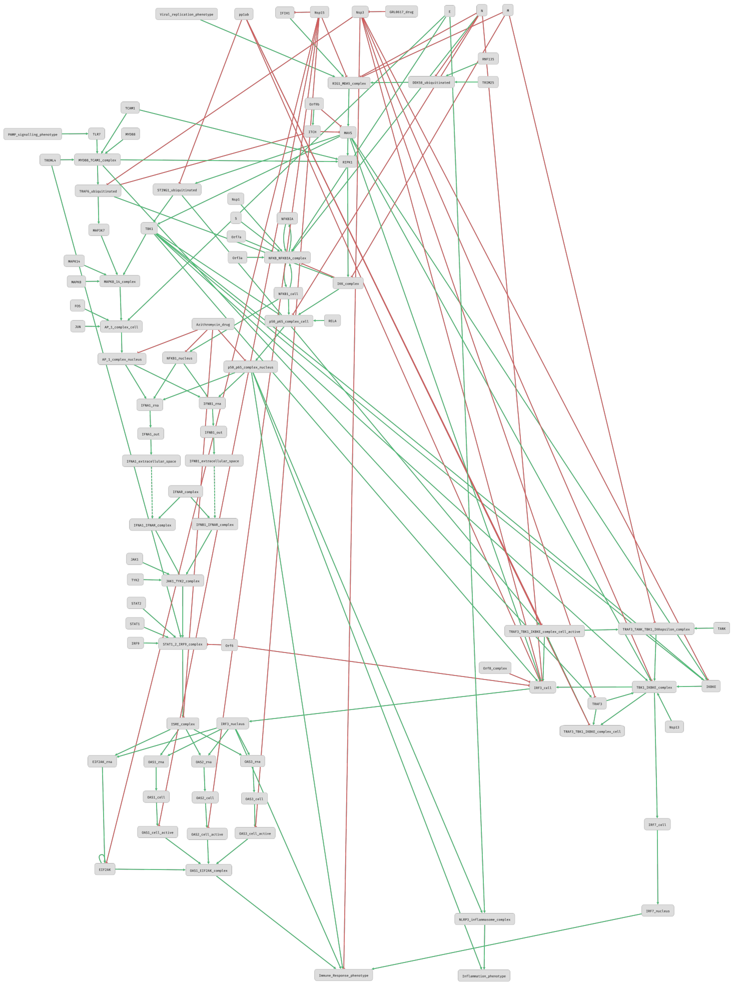

Figure 1: Regulatory graph of the original interferon model.

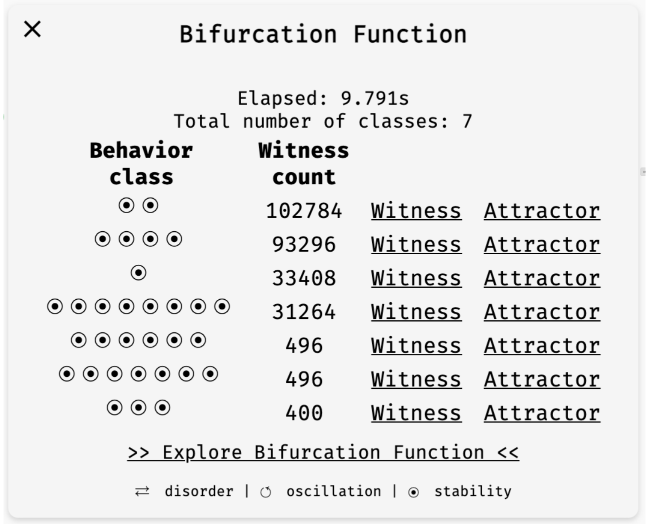

Figure 2: A tabular summary of the bifurcation function of the original model as produced by AEON.

the attractor states of the original model, we can see that in many cases, the values of phenotype-corresponding variables are the same. Furthermore, the attractors often only differ in the values of the three variables in this complex. Here, we will therefore try to eliminate this source of multi-stability.

Overall, the complex has several associated input variables – some are unique to the complex, some are shared with other parts of the model. However, it only interacts with the rest of the model "downstream" using two regulations: `NFKB1-cell` $\rightarrow$ `NFKB1_nucleus` and `NFKB1-cell` $\rightarrow$ `p50_p65_complex_cell`. Consequently, in every network attractor, the behaviour of the model depends on the behaviour of the `NFKB1-cell` variable, as the remaining variables in this complex do not regulate anything else directly.

We can therefore construct a simplified model that only contains this complex and variables on which it depends (available as `models/complex.aeon` and `models/complex.sbml`). By analysing this simplified model in AEON, we can see that the variable `NFKB1-cell` is always stable. Depending on the input variables, it can stabilise as `false`, or show a switched bi-stable behaviour between `true` and `false`. However, an attractor of this fragment of the network will never exhibit unstable behaviour in `NFKB1-cell`, i.e. there is no attractor in which the value of `NFKB1-cell` changes.

As a result, we can replace `NFKB1-cell` with an artificial constant input variable and eliminate the rest of the complex and its inputs (unless they also influence other parts of the network). This yields a reduced model (available as `models/reduced.sbml` and `models/reduced.aeon`). The regulatory graph of this model is shown in Figure 3. A tabular summary of the bifurcation function

of this model is then shown in Figure 4. As we can see, the number of unique stable attractors has decreased considerably compared to Figure 2.

5 Bifurcation Analysis

The bifurcation decision tree as presented in the paper is shown in Figure 5, and a fully expanded version of the tree is shown in Figure 6.

The construction of the tree has been conducted by using the "Explore Bifurcation Function" procedure of AEON. As a root of the constructed BDT, we have selected the input parameter `Viral_dsDNA_rna_reduced` that represents the internal biological phenotype of the cell displaying the detected replication of the virus DNA inside of the cell. Both subtrees have similar distributions of the four attractor phenotypes shown in Figure 4.

The bifurcation decisions in the next level of the tree are based on the input parameter that gives the most significant information gain regarding bifurcation. Interestingly, this parameter reflects the presence/absence of the drug GRL0617. Setting of GRL0617 significantly changes the list of attractor phenotypes. Despite the setting of `Viral_dsDNA_rna_reduced`, non-presence of GRL0617 results in a single stable attractor or a bistable attractor, respectively, depending on the presence (resp. absence) of the virus protein `Nsp15`.

In the case where GRL0617 is present, we have selected the well known azithromycin antibiotics drug as the next input parameter decision point. Although azithromycin has been very recently clinically reported as not significant for COVID treatment, we have been interested in how it affects the distribution of the attractors. The drug is known for its anti-inflammatory effects. In this model, azithromycin gives very low information gain towards bifurcations. Anyway, the corresponding subtrees with different settings of the root `Viral_dsDNA_rna_reduced` have different structure. We have proceeded in manually generating the subtrees by following the same order of parameter decisions. All of these parameters correspond to the virus proteins with the only exception of `TREML4` (the signalling protein reporting the presence of a virus in the organism) and `NFKBIA_NFKB_component` (the "virtual" parameter introduced during the model reduction procedure).

6 Phenotype Expression

AEON allows to display detailed information concerning values of variables in attractors of the selected class/node of the BDT. In particular, by running the task "Stability Analysis" for a selected node of the BDT, the list of all model variables is reported including the values they reach in attractors corresponding with the BDT node. In particular, for every variable it is reported for how many parametrisations it appears as true (resp. false) in a single stable state attractor, and in how many parametrisations it is bistable (true and false in different stable states of a multi-stable attractor). Indirectly, the stability analysis over

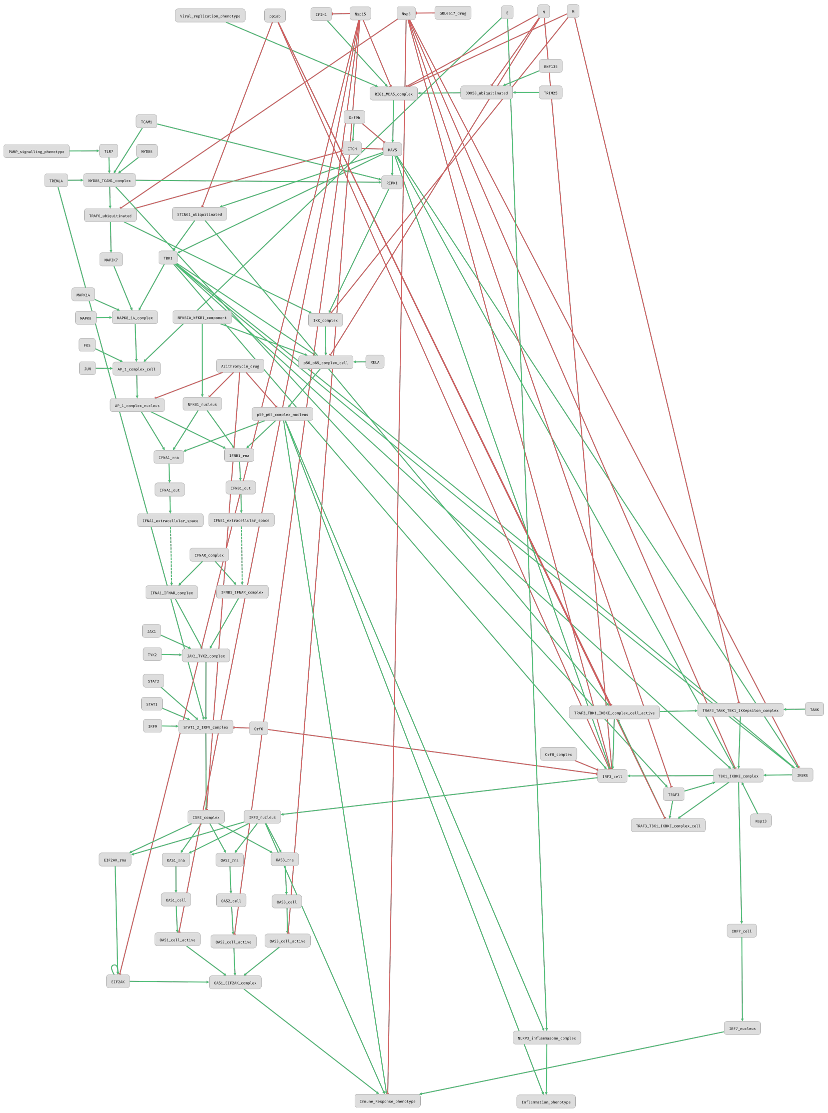

Figure 3: Regulatory graph of the reduced interferon model.

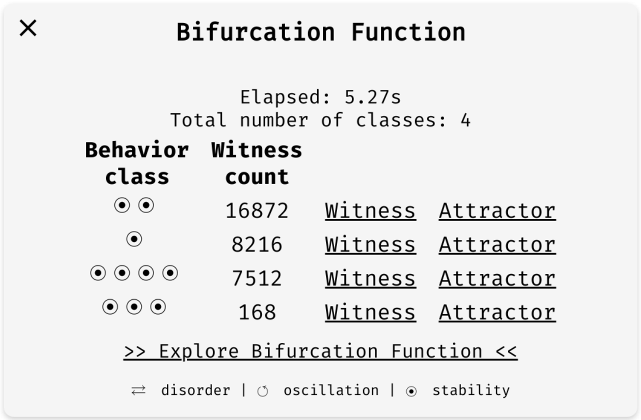

Figure 4: A tabular summary of the bifurcation function of the reduced model as produced by AEON.

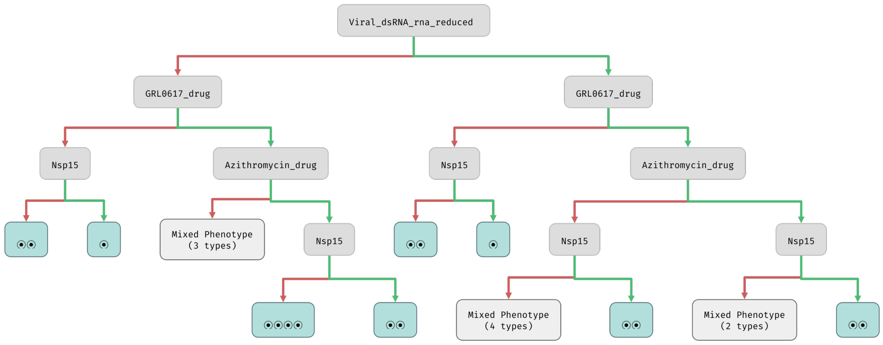

Figure 5: Bifurcation decision tree of the reduced model as presented in the paper.

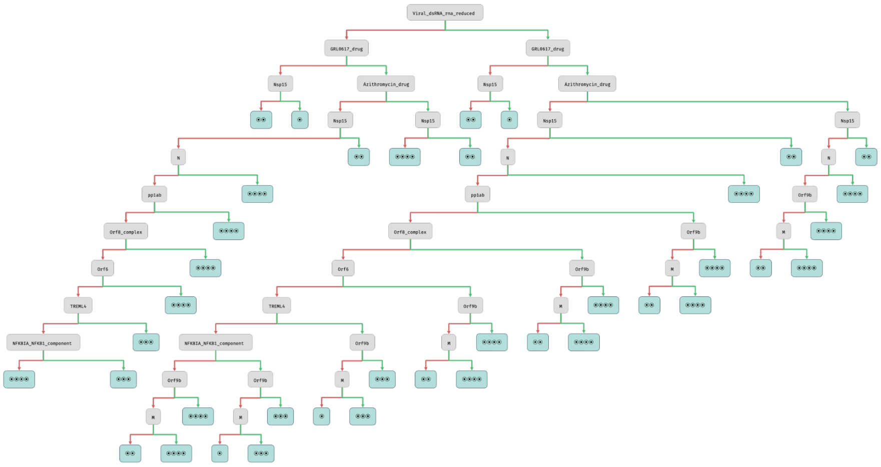

Figure 6: A fully expanded bifurcation decision tree of the reduced model (a high resolution version available as `figures/tree_full.png`).

the parameterised BN gives us the information on how robust the stability of the given variable is with respect to perturbations in the parameters.

In the case study model, we have used the stability analysis to assess the long-term behaviour of the variables representing the individual biological phenotypes. This information is presented in Table 1 (also shown in the main paper). In particular, the effect of setting GRL0617 to false always (in the context of the constructed BDT) results in fixing the immune response to false. On the contrary, the presence of GRL0617 enables the possibility of getting the immune response bistable or fixing it to true. Assuming GRL0617 set true, another significant observation is that after setting azithromycin true the biological phenotype corresponding to the production of interferons is fixed to false. Finally, the paths of the BDT where both GRL0617 and azithromycin are true display an important decision point caused by the Nsp15 virus protein. In particular, this virus protein disables the immune response to appear true on a single stable attractor. The effect of this protein on the immune response is therefore destabilising.

Every row of Table 1 has been constructed by employing the stability analysis in a simple BDT where the studied parameter (component) is chosen at the root. In particular, we obtain the information on how the behaviour of phenotype-related variables in attractors changes with setting the studied parameter to true. More specifically, for each biological phenotype, we compute the portion of parametrisations where it is true at a single steady state and where it is bistable. The reported trend characterises how this portion changes with respect to the situation where all parameters (including the root) are not fixed. In Figure 7 there are depicted the respective BDTs used for the biological

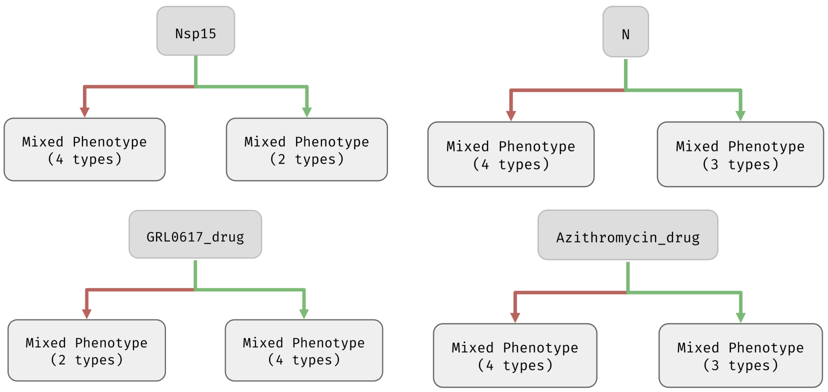

Figure 7: Rooted decisions over selected input parameters. Stability analysis in mixed phenotype nodes makes the source of data used to generate Table 1.

**component**	**interf. production**		**IIR**		**INFL**	
	$\odot$	$\odot\odot$	$\odot$	$\odot\odot$	$\odot$	$\odot\odot$
N	↘	↘	↘	↗	↘	↗
Nsp15	—	—	↘	↗	—	—
GRL0617	—	↗	↗	↗	—	↗
azithromycin	↘	↗	↘	↗	↘	↗

Table 1: Qualitative influence of individual components on the stabilisation (more/less prominent or unchanged) of a particular phenotype (column) in either stable ($\odot$) or bistable ($\odot\odot$) regime.

phenotype stability analysis.

The exact numeric data based on which Table 1 is constructed is available in `tables/stability_analysis.txt`. In this file, we have four smaller tables, each showing the distribution of phenotypes in different situation. First, a general case (all parameters unknown) gives us a baseline. Then, for each of the considered parameters (N, Nsp15, GRL0617, and azithromycin), a table shows the distribution of phenotypes when a parameter is fixed to true. Based on this, a "trend" in Table 1 can be inferred.

References

[1] Jiří Barnat et al. Detecting attractors in biological models with uncertain parameters. In *Computational Methods in Systems Biology*, pages 40–56, Cham, 2017. Springer.

[2] Nikola Beneš et al. Formal analysis of qualitative long-term behaviour in parametrised Boolean networks. In *International Conference on Formal Engineering Methods*, pages 353–369, Cham, 2019. Springer.

[3] Nikola Beneš et al. AEON: Attractor bifurcation analysis of parametrised Boolean networks. In *Computer Aided Verification*, pages 569–581, Cham, 2020. Springer.

[4] Nikola Beneš et al. Computing bottom SCCs symbolically using transition guided reduction. In *International Conference on Computer Aided Verification*, pages 505–528, Cham, 2021. Springer.

[5] Randal E. Bryant. Graph-based algorithms for Boolean function manipulation. *IEEE Trans. Computers*, 35(8):677–691, 1986.

[6] J. Ross Quinlan. Induction of decision trees. *Machine learning*, 1(1):81–106, 1986.

[7] Yang Zhao and Gianfranco Ciardo. Symbolic computation of strongly connected components and fair cycles using saturation. *Innovations in Systems and Software Engineering*, 7(2):141–150, 2011.
